# TLR2^−/−^ Mice Display Increased Clearance of Dermatophyte *Trichophyton mentagrophytes* in the Setting of Hyperglycemia

**DOI:** 10.3389/fcimb.2017.00008

**Published:** 2017-01-20

**Authors:** Débora de Fátima Almeida, Thais F. de Campos Fraga-Silva, Amanda R. Santos, Angela C. Finato, Camila M. Marchetti, Marjorie de Assis Golim, Vanessa S. Lara, Maria S. P. Arruda, James Venturini

**Affiliations:** ^1^Laboratory of Experimental Immunopathology, Department of Chemistry, Universidade Estadual Paulista Bauru, Brazil; ^2^Department of Microbiology and Immunology, Institute of Biosciences of Botucatu, Universidade Estadual Paulista Botucatu, Brazil; ^3^Botucatu Blood Center, Universidade Estadual Paulista Botucatu, Brazil; ^4^Department of Surgery, Stomatology, Pathology and Radiology, Bauru School of Dentistry, University of São Paulo Bauru, Brazil

**Keywords:** toll-like receptors, hypoinsulinemia-hyperglycemia, *Trichophyton mentagrophytes*, deep dermatophytosis, monocytes

## Abstract

Dermatophytosis is one of the most common human infections affecting both immunocompetent individuals and immunocompromised patients, in whom the disease is more aggressive and can reach deep tissues. Over the last decades, cases of deep dermatophytosis have increased and the dermatophyte-host interplay remains poorly investigated. Pattern recognition molecules, such as Toll-like receptors (TLR), play a crucial role against infectious diseases. However, there has been very little research reported on dermatophytosis. In the present study, we investigated the role of TLR2 during the development of experimental deep dermatophytosis in normal mice and mice with alloxan-induced diabetes mellitus, an experimental model of diabetes that exhibits a delay in the clearance of the dermatophyte, *Trichophyton mentagrophytes* (Tm). Our results demonstrated that inoculation of Tm into the footpads of normal mice increases the expression of TLR2 in CD115^+^Ly6C^high^ blood monocytes and, in hypoinsulinemic-hyperglycemic (HH) mice infected with Tm, the increased expression of TLR2 was exacerbated. To understand the role of TLR2 during the development of murine experimental deep dermatophytosis, we employed TLR2 knockout mice. Tm-infected TLR2^−/−^ and TLR2^+/+^ wild-type mice exhibited similar control of deep dermatophytic infection and macrophage activity; however, TLR2^−/−^ mice showed a noteworthy increase in production of IFN-γ, IL-10, and IL-17, and an increased percentage of splenic CD25^+^Foxp3^+^ Treg cells. Interestingly, TLR2^−/−^ HH-Tm mice exhibited a lower fungal load and superior organization of tissue inflammatory responses, with high levels of production of hydrogen peroxide by macrophages, alongside low TNF-α and IL-10; high production of IL-10 by spleen cells; and increased expansion of Tregs. In conclusion, we demonstrate that TLR2 diminishes the development of adaptive immune responses during experimental deep dermatophytosis and, in a diabetic scenario, acts to intensify a non-protective inflammatory response.

## Introduction

Dermatophytosis is one of the most frequent forms of human infections. It is estimated that the disease affects around 20–25% of world population and the incidence has increased continuously (Havlickova et al., 2008). Dermatophytes are classified in three genera: *Microsporum, Trichophyton*, and *Epidermophyton*, and the species *Trichophyton rubrum* and *Trichophyton mentagrophytes* (Tm) are the most frequent etiological agents in humans (Vena et al., 2012). Over the last decades, cases of deep dermatophytosis have increased, particularly in immunocompromised patients, such as patients with solid organ transplantation, hematological malignancy, immunosuppressive therapy, CARD9 deficiency, and diabetes mellitus (Marconi et al., 2010; Lanternier et al., 2013). Deep dermatophytosis is characterized by lesions beyond the perifollicular area and the fungi can invade the bloodstream and disseminate to internal organs, including the lymph nodes, brain, liver, muscle, and bone (Marconi et al., 2010).

Although clinical and experimental studies have indicated the relevance of T cells in resistance to dermatophytes (Calderon and Hay, 1984; Miyata et al., 1996; Almeida, 2008; Venturini et al., 2012), keratinocytes (Grappel et al., 1974), neutrophils (Szepes et al., 1993), and macrophages (Campos et al., 2006) are also involved in the immunology of dermatophytosis. Despite its importance, the interaction between phagocytes and dermatophytes has rarely been studied. Campos et al. (2006) observed that *T. rubrum* exoantigens and mannan are able to inhibit the phagocytosis of conidia, down-regulate the expression of MHC-II, and induce the production of IL-10 by peritoneal adherent cells (PACs).

The recognition of fungi by toll-like receptors (TLRs) has emerged as an important step in triggering a regulated and efficient inflammatory response. However, the participation of TLR2 in particular in fungal diseases, and the outcomes of immune responses, is varied. For instance, TLR2 dependent mechanisms induced by *Candida albicans* (Netea et al., 2004) contribute to the evasion or inhibition of immune responses stimulated by increased IL-10 production and survival of regulatory T cells (Tregs) (Netea et al., 2004). In contrast, TLR2 has been associated with host protection from *Penicillium marneffei* (Nakamura et al., 2008) and *Cryptococcus neoformans* (Yauch et al., 2004). The participation of TLR2 in dermatophytosis has been evaluated in keratinocyte cell culture and it was demonstrated that *T. rubrum* conidia suppress the expression of surface TLR2 on keratinocytes after 24 h (Huang et al., 2015); according to these authors, suppression of TLR2 expression acts as an immune escape mechanism.

Experimental models of dermatophytosis employ guinea pigs (Saunte et al., 2008) but dermatophytosis in these species are restricted, being difficult the investigation of the aspects of the disseminated infection. In order to overcome these claims other authors have been study the dermatophytosis using murine models (Campos et al., 2006; Venturini et al., 2012; Fraga-Silva et al., 2015; Yoshikawa et al., 2016). Recently, Yoshikawa et al. (2016), using a murine model of deep dermatophytosis with lack of Dectin-1 and/or Dectin-2, demonstrated that absence of these C-type lectin receptors promotes an inefficient pro-inflammatory response against *T. rubrum* infection characterized by lower production of TNF-α and IL-1β by spleen cells and impairing the dermatophytosis resolution compared to control mice (Yoshikawa et al., 2016). Our group have also studied aspects of the immune response in deep experimental dermatophytosis, by subcutaneous inoculation of Tm into the footpads of Swiss mice (Venturini et al., 2012). In this murine model, fungal inoculation results in an initial exudative lesion in the local area of the injection and disseminated infection to internal organs. After 14 days, the fungal load decreases and a typical Th1 response is organized, characterized by a positive delayed-type hypersensitivity test and a granulomatous tissue reaction involving epithelioid cells (Venturini et al., 2012). Furthermore, we have employed alloxan-induced hypoinsulinemic-hyperglycemic (HH) mice (Venturini et al., 2011; Fraga-Silva et al., 2015) to evaluate the immune response in a susceptible host, since patients with diabetes mellitus exhibit a higher incidence of dermatophytosis and the disease is usually more severe (Eckhard et al., 2007; Kim et al., 2016). Delayed fungal clearance is observed in HH-infected mice (Venturini et al., 2011). In the present study, we investigated the role of TLR2 during the development of Tm infection in naïve and HH mice to better understand the initial events in deep dermatophytosis.

## Materials and methods

### Mice

Male Swiss and C57BL/6 mice were purchased from CEMIB/UNICAMP (Campinas, São Paulo, Brazil) and TLR2 knockout mice (C57BL/6 TLR2^−/−^) were purchased from the University of São Paulo (USP, Ribeirão Preto, São Paulo, Brazil). All mice were 45 days old at the time of purchase and received a sterile balanced diet, water *ad libitum* and were kept in a ventilated shelf ALERKS-56 housing system (Alesco®, Monte Mor, São Paulo, Brazil). The experimental protocol was performed in accordance with the ethical principles for animal research adopted by the National Council for the Control of Animal Experimentation (CONCEA). This study was approved by the Ethical Committee of School of Sciences (Proc. 1269/46/01/10, UNESP, Bauru, São Paulo, Brazil).

### Experimental design

Mice from each strain were allocated into four groups: group Tm was composed of Tm-infected mice; group HH-Tm of HH-induced, Tm-infected mice; group HH of HH-induced mice; and group CTRL of mice with normal insulin and glycaemia, that were subjected to the same inoculation procedures using sterile saline solution (SSS).

### Alloxan administration

The HH condition was induced by alloxan administration, which selectively destroys the insulin-producing pancreatic beta-cells (Huang and Wu, 2005; Lenzen, 2008). Mice were intravenously inoculated with alloxan in the caudal vein with a single dose of 60 mg/kg of body weight (Sigma-Aldrich Co., St. Louis, MO, USA). Hyperglycemia was confirmed 48 h later. Only mice showing blood glucose levels greater than 200 mg/dl were included in the study. The destruction of pancreatic beta-cells and the absence of pancreatitis were confirmed by histological analyses (data not shown) (Rossini et al., 1977; Abd elaziz, 2011). Control mice without HH (Tm and control groups) were inoculated in the caudal vein with SSS and had blood glucose levels ranging from 90 to 130 mg/dl.

### Tm inoculum

Tm strain (2118/99-ILSL) was originally obtained from the fungal collection of Lauro de Souza Lima Institute (Bauru, São Paulo, Brazil) and maintained on Mycosel® agar slants (Difco Laboratories, Detroit, Michigan, USA). For infection of mice, Tm was cultured in the same media for 10 days at 25°C, washed carefully with SSS, the suspension mixed twice for 10 s on a vortex-mixer, and decanted for 5 min. Supernatants were collected and washed twice. Fungal viability was determined by cotton blue staining, and concentrations adjusted to 5 × 10^8^ viable Tm conidia/ml (Venturini et al., 2012). Mice were subcutaneously injected in the right footpad with 0.04 ml of Tm inoculum (2 × 10^7^ conidia).

### Collection of biological samples

Mice from each group were randomly selected and euthanized by CO_2_ inhalation. Peritoneal cells were collected by washing the peritoneal cavity with 10 ml of ice-cold sterile phosphate buffered saline (PBS), pH 7.4. Footpads and spleens were collected aseptically. Blood samples were collected via intracardiac puncture using EDTA as an anticoagulant.

### Determination of fungal load

Footpad samples were weighed and macerated in 1.0 ml of SSS and 100 μl was spread over culture plates containing Mycosel agar® (Difco) using a Drigalski T loop. The plates were sealed and incubated at 25°C for 7 days. The number of colony forming units (CFU) was normalized per gram of tissue and log transformed.

### Histological procedures

Fragments of footpad were removed and fixed in 10% neutral buffered formalin. Paraffin-embedded sections (5 μm) were stained with hematoxylin and eosin (HE) and analyzed using an optical microscope (Leica DM 750); images were acquired using a digital camera (Leica ICC50 HD) attached to the microscope.

### FACS analysis

Spleen cells and peripheral blood cells were collected and the red blood cells lysed with buffer containing NH_4_Cl. Four-color staining was conducted using combinations of fluorochrome-conjugated monoclonal anti-mouse antibodies (eBioscience, San Diego, CA, USA) as follows: CD3-PerCP (clone 145-2C11), CD4-FITC (clone GK1.5), CD25-APC (clone PC61.5), and Foxp3-PE (clone FJK-16s) for splenic T regulatory cells, and TLR2-PE (clone 6C2), CD115-APC (clone AFS98), and Ly6C-PercP (clone HK1.4) for monocyte subsets. Analysis was performed using a FACSCalibur™ Flow Cytometry System (BD Biosciences, San Jose, CA, USA), at the Botucatu Blood Center (UNESP, Botucatu, SP, Brazil), and data were analyzed using FlowJo software (TreeStar, Ashland, OR, USA).

### Peritoneal adherent cell (PAC) culture

To evaluate macrophage activity, we performed an *in vitro* PAC assay. Peritoneal cells were collected with PBS, the suspension centrifuged, and cells resuspended in 1.0 ml of RPMI-1640 (Nutricell, Campinas, SP, Brazil) supplemented with 10% heat-inactivated fetal calf serum (Gibco BRL, Grand Island, NY, USA), penicillin (100 IU/ml), and streptomycin (100 mg/ml) (Gibco). The cell concentration was adjusted to 1.0 × 10^6^ mononuclear phagocytes/ml as determined by the uptake of 0.02% neutral red. Peritoneal cells were aliquoted into 96-well flat-bottomed microtiter plates (0.1 ml/well) (Costar, Cambridge, MA, USA) and incubated for 2 h at 37°C, 5% CO_2_ in a humidified chamber to allow peritoneal cells to adhere and spread. Non-adherent cells were removed by washing the wells three times with RPMI-1640, and the remaining PACs (>95% mononuclear phagocytes as assessed by morphological examination and expression of F4/80 by FACS) were used for experiments. PACs were cultured in RPMI-1640 medium, or RPMI-1640 medium supplemented with 50 μg/ml Tm exoantigen (TmExo), produced as described in Venturini et al. (2008). After 24 h, cell-free supernatants were harvested and stored at −80°C for cytokine analysis.

### Hydrogen peroxide (H_2_O_2_) release

After cell culture incubation, supernatants were removed and PACs incubated with phenol red solution [dextrose (Sigma), phenol red (Sigma), horseradish peroxidase type II (Sigma)] and plated at 37°C, 5% CO_2_ for 1 h, according to Russo et al. (1989). The reaction was stopped by the addition of 1 M NaOH and the H_2_O_2_ concentration determined using an ELISA microreader (EL800, BIO-TEK Instruments, Inc.). The concentration was calculated using an analytical curve (0.5–8.0 nM H_2_O_2_).

### Spleen cell culture

Fragments of spleen were collected and homogenized in ice-cold PBS. The red blood cells were lysed with buffer containing NH_4_Cl, and the remaining cells washed with RPMI. After washing, the suspension was centrifuged, and the cells resuspended in 1 ml of complete medium. The concentration was adjusted to 5.0 × 10^6^ cells/mL, as determined by 0.1% trypan blue staining. Afterwards, the cells were challenged with TmExo (50 μg/ml) or medium. The culture was incubated at 37°C, 5% CO_2_ in a humidified chamber for 48 h. Cell-free supernatants were harvested and stored at −80°C for cytokine analysis.

### Cytokine analyses

The levels of IL-10, IFN-γ, TNF-α, IL-17, and IL-1β were measured in cell-free supernatants using a cytokine Duo-Set Kit (R&D Systems, Minneapolis, MI, USA), according to the manufacturer's instructions. The results were expressed in pg/mL, and determined from standard curves established for each assay.

### Statistical analyses

Statistical tests were performed using GraphPad Prism 5.0 (GraphPad Software. Inc., San Diego, California, USA), and the significance level established to verify the null hypothesis was 5.0%. The data passed a normality test (Shapiro-Wilk) and comparisons of two independent samples were performed using *t*-tests for parametric samples (Zar, 2010).

## Results

### *T. mentagrophytes* infection induces TLR2 expression in granulocytes and monocyte subsets

**Figure 1:**
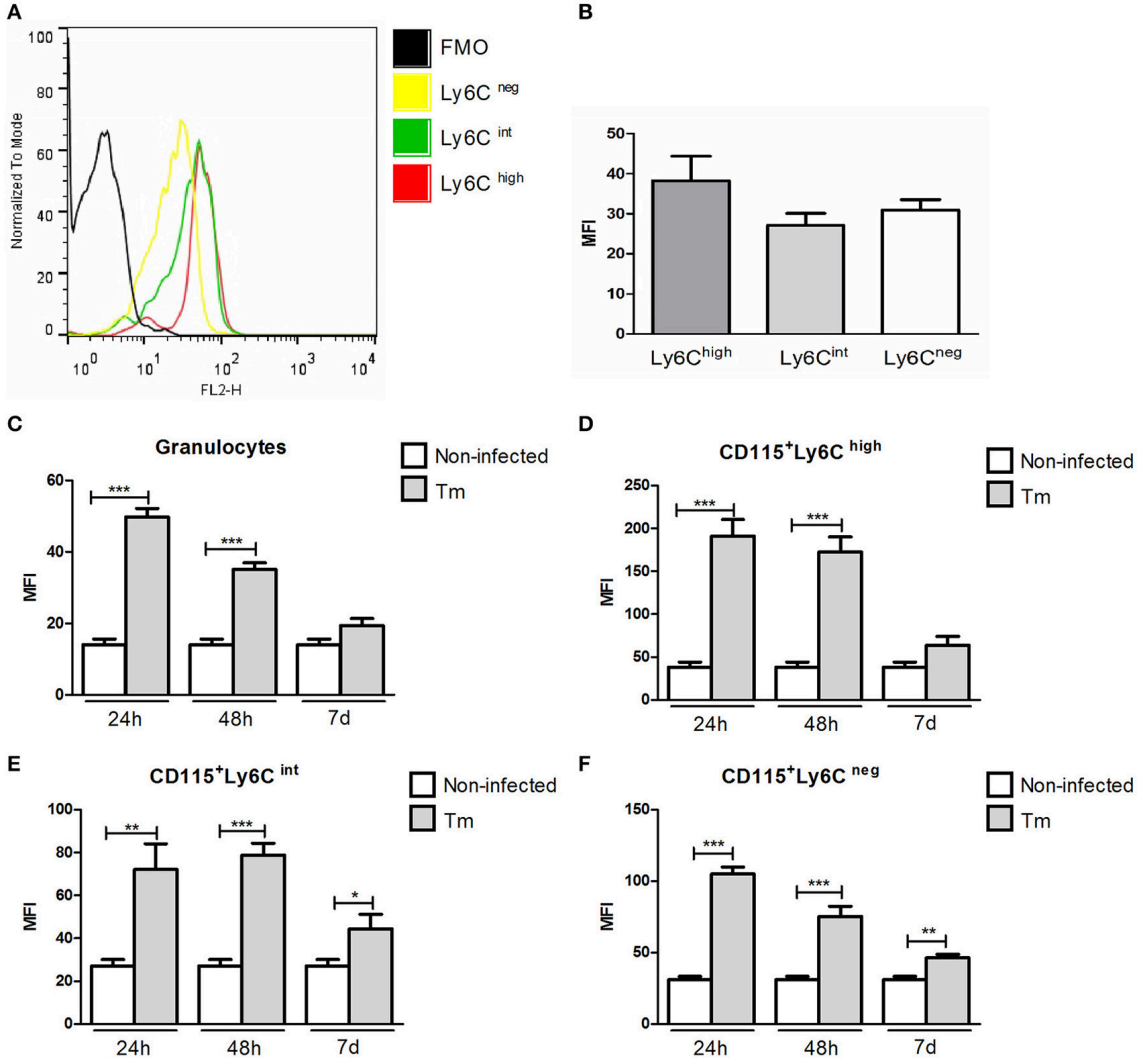
**Expression of TLR2 in peripheral blood granulocytes and monocyte subsets from Swiss mice**. Histogram **(A)** and mean fluorescence intensity **(B)** of TLR2 expression in monocyte subsets of naïve mice. Expression of TLR2 in granulocytes **(C)** and monocyte subsets **(D–F)** of non-infected and Tm-infected mice, evaluated at 24 and 48 h, and 7 days after infection. The results are expressed as means ± SEM (Unpaired *t*-test; ^*^P < 0.05, ^**^P < 0.01, ^***^P < 0.001; *n* = 5–6).

The levels of TLR2 were similar among monocyte subsets (CD115^+^Ly6C^high^, CD115^+^Ly6C^int^, and CD115^+^Ly6C^neg^) in Swiss mice under baseline line conditions (Figures 1A,B).

In Tm-infected mice (non-diabetic), we observed higher of levels of TLR2 expression in granulocytes (Figure 1C) and inflammatory monocytes (CD115^+^Ly6C^high^) (Figure 1D) at 24 and 48 h post-infection (p.i.), compared with non-infected mice. The expression of TLR2 by intermediate (CD115^+^Ly6C^int^) and resident (CD115^+^Ly6C^neg^) blood monocytes was also higher in Tm-infected, than non-infected, mice at 24 and 48 h, and 7 days, p.i. (Figures 1E,F).

### During infection with *T. mentagrophytes*, TLR2 diminishes the polarization of the Th1 and Th17 immune responses and decreases the proportion of Tregs in the spleen

To evaluate the involvement of TLR2 in experimental dermatophytosis, we employed Tm-infected TLR2^+/+^ and TLR2^−/−^ C57BL/6 mice. Although Swiss mice have been considered naturally more susceptible to Tm (Schmitt et al., 1963; Fischman et al., 1976), we observed that Tm-infected C57BL/6 mice exhibited slight higher fungal loads 24 h p.i. than Swiss mice and, as observed in Swiss mice, the fungal load on day 7 p.i. was decreased in C57BL/6 mice (Supplementary Figure 1). Therefore, the development of experimental Tm infection is not influenced by differences in the genetic backgrounds between these strains.

**Figure 2:**
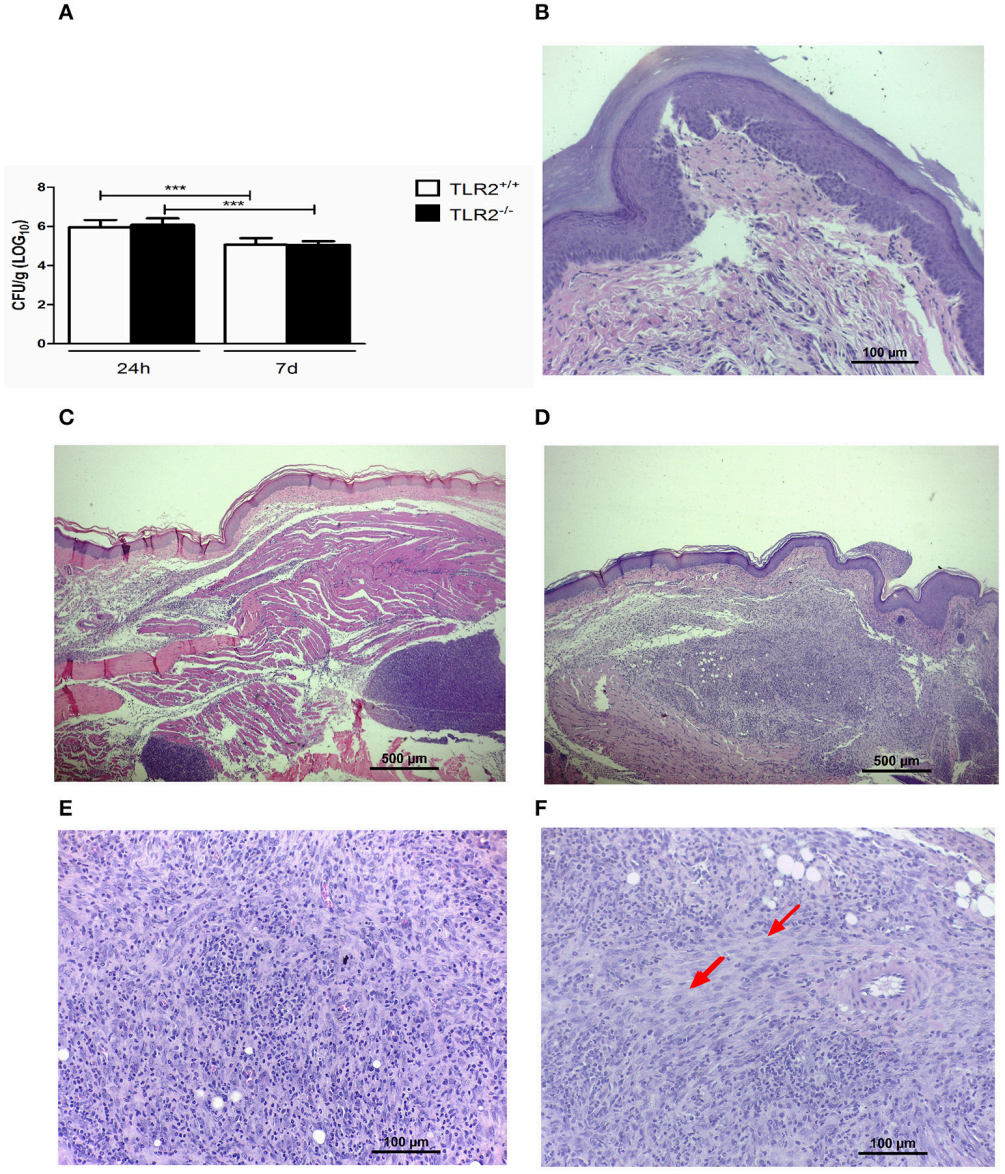
**The dynamics of fungal infection in Tm-infected TLR2^**+/+**^ and TLR2^**−/−**^ mice. (A)** Recovery of viable fungi from footpads of Tm-infected mice 24 h and 7 days p.i. Results are expressed as mean ± SD (Unpaired *t*-test: ^***^P < 0.001). **(B)** Footpad of a naïve mouse (HE). **(C)** Footpad of a TLR2^+/+^ mouse 24 h after inoculation, showing a typical acute inflammatory response, characterized by an influx of neutrophils, edema, and abscess formation (HE). **(D)** Footpad of a TLR2^+/+^ mouse 7 days after infection, showing an initial granulomatous reaction with a small number of neutrophils and modified macrophages; details are shown in **(E)** (HE). **(F)** Footpad of a TLR2^−/−^ mouse 7 days after infection, showing a well-organized granulomatous response, characterized by modified macrophages and epithelioid cells (arrow) (HE).

We observed no difference between Tm-infected TLR2^−/−^ and TLR2^+/+^ mice in the amount of viable fungi recovered from inoculated footpads (Figure 2A). Moreover, histopathological investigation (Figures 2B–F) demonstrated the same profile in both groups at 24 h p.i.; the lesions showed a typical acute inflammatory response, characterized by an influx of neutrophils, edema, and abscess formation (Figure 2C). On day 7, TLR2^+/+^ mice exhibited an initial granulomatous reaction (Figure 2D), involving macrophages and a small number of neutrophils (Figure 2E). In Tm-infected mice TLR2^−/−^, granulomas were better organized and epithelioid cells were also observed (Figure 2F). Next, we evaluated the immunological responses of these mice.

**Figure 3:**
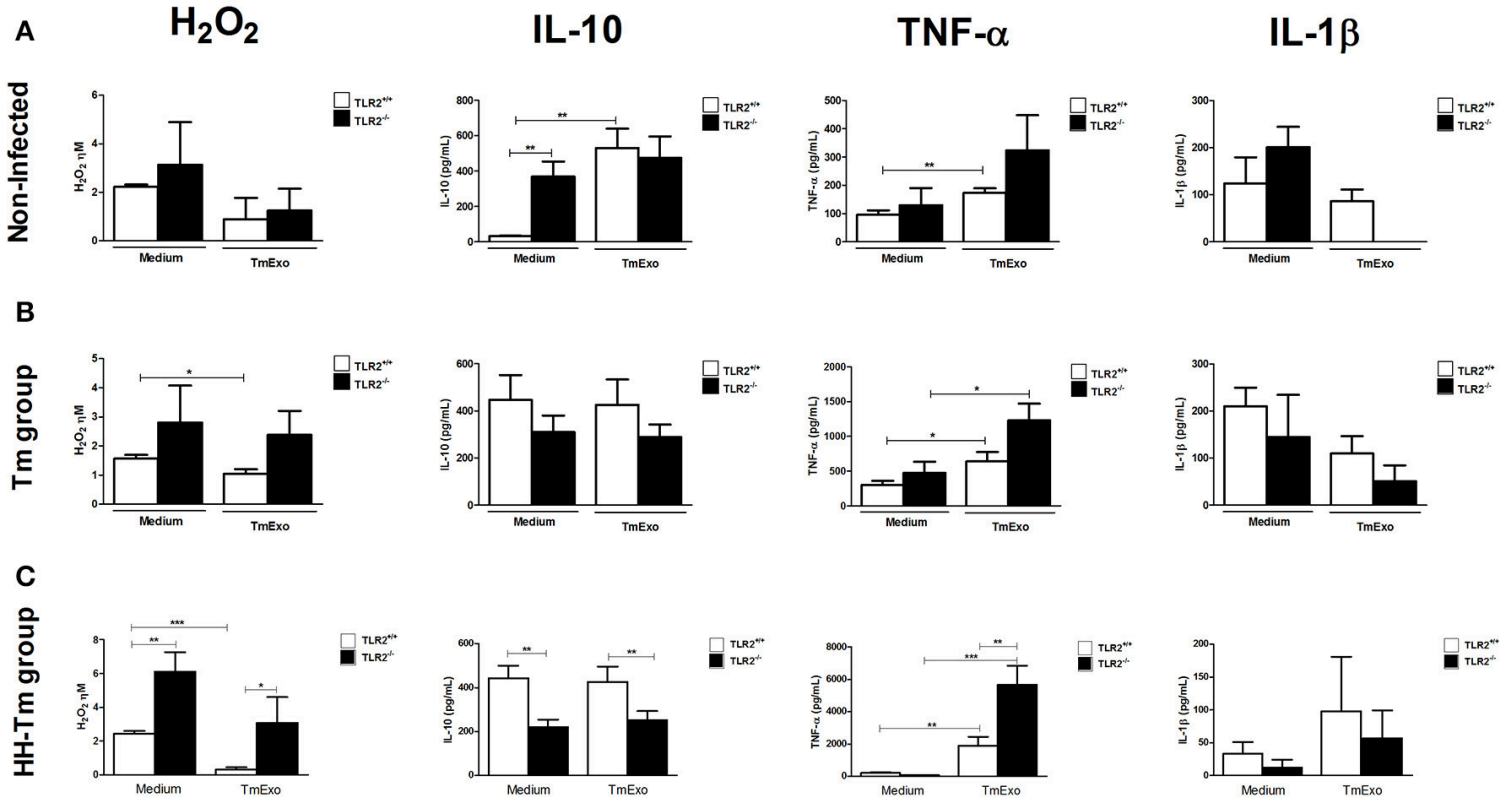
**Macrophage activity in naïve, Tm-infected, and HH-Tm TLR2^**+/+**^ and TLR2^**−/−**^ mice**. Peritoneal adherent cells were cultured in the presence or absence of TmExo and, after 24 h, the levels of hydrogen peroxide (H_2_O_2_), TNF-α, and IL-1β were determined in cell-free supernatants. **(A)** TLR2^+/+^ and TLR2^−/−^ naïve mice, **(B)** Tm-infected TLR2^+/+^ and TLR2^−/−^ mice, **(C)** HH-Tm TLR2^+/+^ and TLR2^−/−^ mice. Results are expressed as means ± SEM. (Unpaired *t*-test; ^*^P < 0.05, ^**^P < 0.01, ^***^P < 0.001; *n* = 5–6).

In order to evaluate the ability of TLR2 to trigger a Tm antigen-specific response, we first compared the macrophage activity in naïve TLR2^−/−^ and TLR2^+/+^ mice and then in the presence and absence of Tm exoantigen (Figure 3). PAC of TLR2^−/−^ mice showed a higher spontaneous production of IL-10 than those of TLR2^+/+^ mice (Figure 3A). In response to Tm antigens, we observed that PACs from TLR2^−/−^ mice did not produce detectable levels of IL1-β, whereas this cytokine was detected in media from TLR2^+/+^ cells (Figure 3A). In Tm-infected mice, PAC from both TLR2^+/+^ and TLR^−/−^ mice showed similar macrophage activity in response to TmExo (Figure 3B).

**Figure 4:**
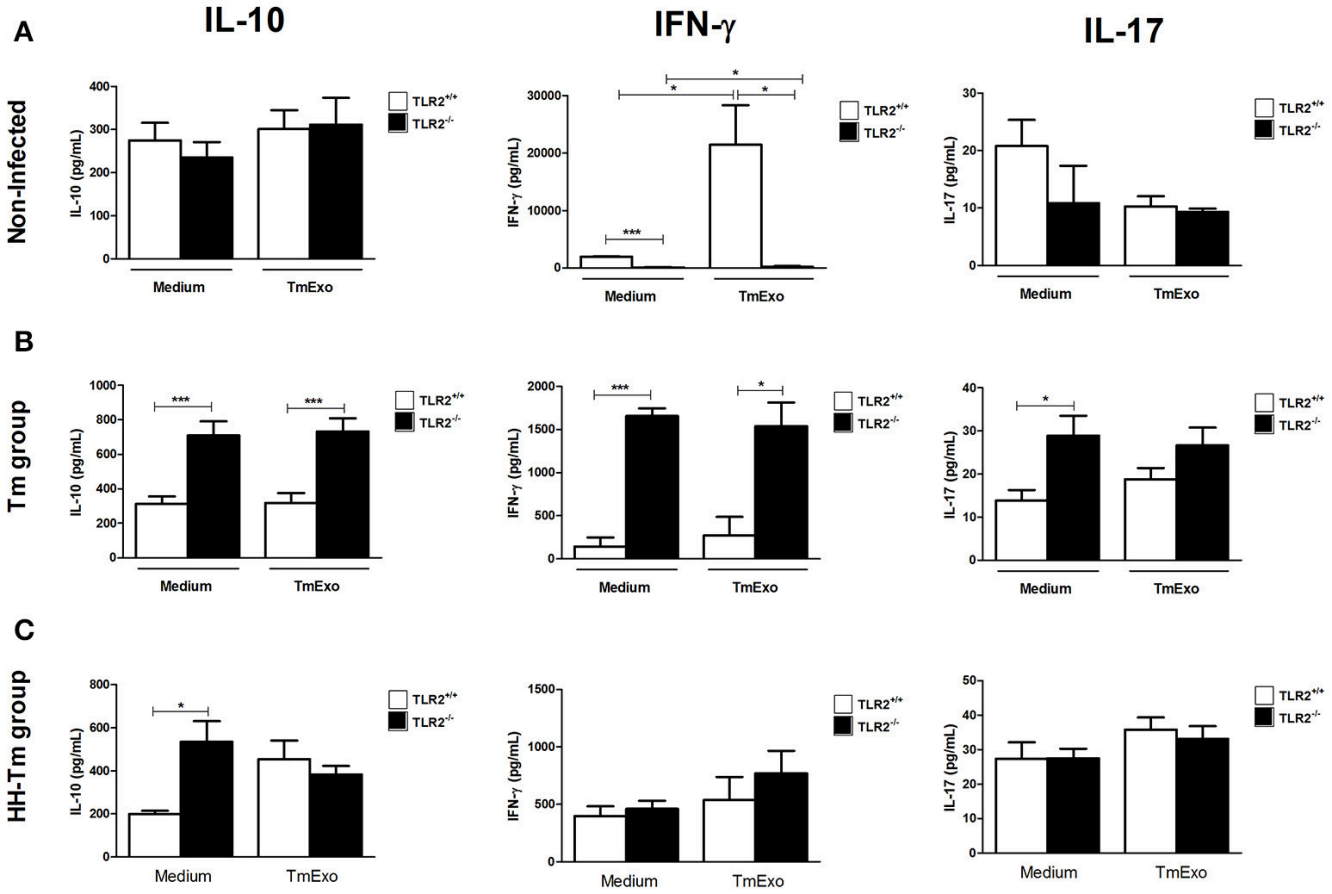
**Activity of splenic cells from naïve, Tm-infected, and HH-Tm TLR2^**+/+**^ and TLR2^**−/−**^ mice**. Spleen cells collected 7 days p.i. were cultured in the presence or absence of TmExo and the levels of IL-10, IFN-γ, and IL-17 were determined in the cell-free supernatants. **(A)** TLR2^+/+^ and TLR2^−/−^ naïve mice, **(B)** Tm-infected TLR2^+/+^ and TLR2^−/−^ mice, **(C)** HH-Tm TLR2^+/+^ and TLR2^−/−^ mice. Results are expressed as means ± SEM (Unpaired *t*-test; ^*^P < 0.05, ^***^P < 0.001; *n* = 5–6).

**Figure 5:**
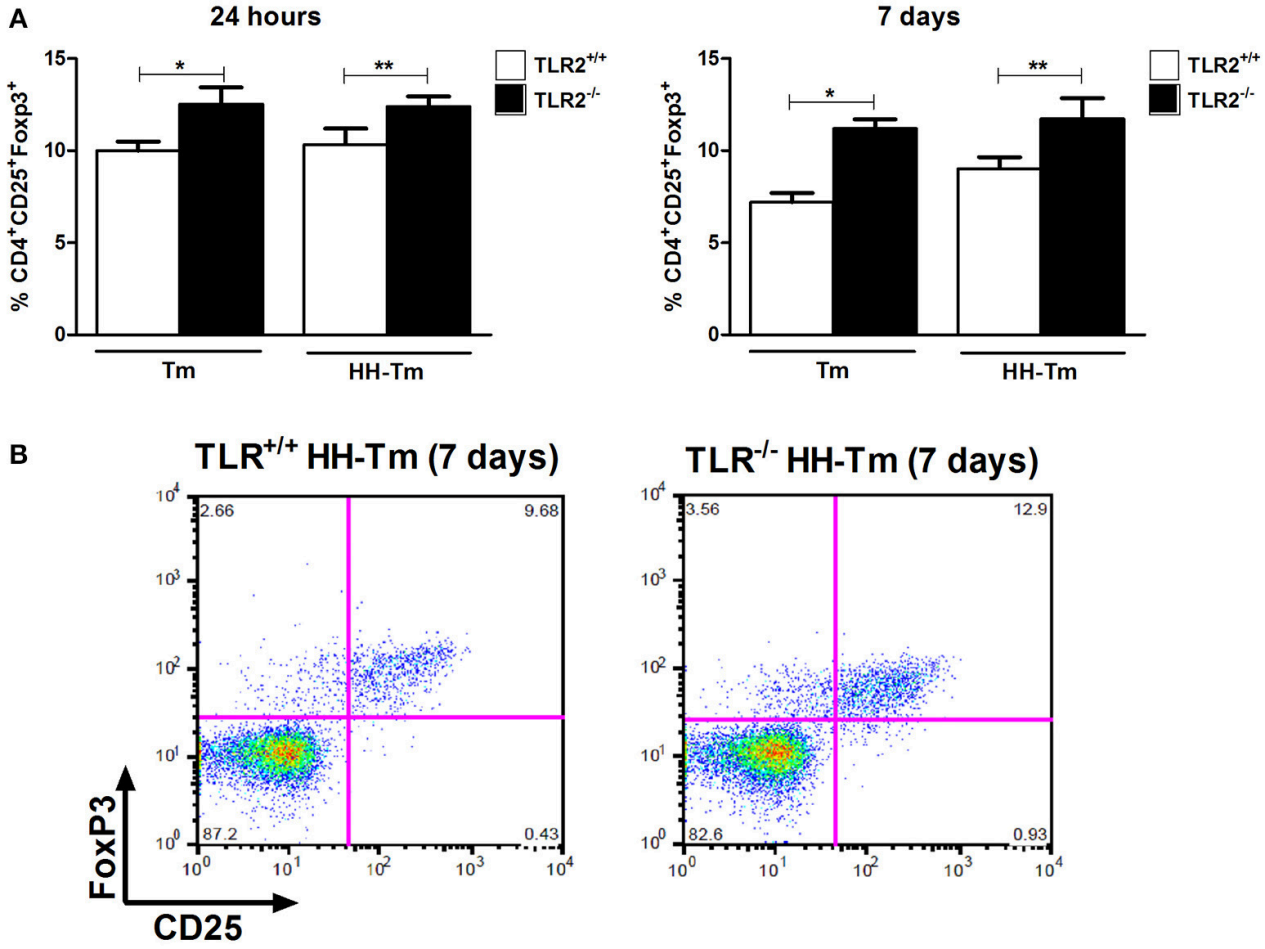
**The distribution of CD25^**+**^Foxp3^**+**^ regulatory T lymphocytes in spleen cells. (A)** Tm and HH-Tm TLR2^+/+^ and TLR2^−/−^ mice were evaluated 24 h and 7 days after infection. Results are expressed as means ± SEM (Unpaired *t*-test: ^*^P < 0.05, ^**^ 0.01 < *P* < 0.05). **(B)** Representative dot plots of the gating strategy for CD25^+^Foxp3^+^.

We next evaluated aspects of the adaptive immune response in spleen cell culture. Naïve TLR2^−/−^ mice produced lower levels of IFN-γ than TLR2^+/+^ mice, independently of stimulus (Figure 4A); however, no differences were observed in the production of IL-10 and IL-17 (Figure 4A). In Tm-infected TLR2^−/−^ mice, spleen cells expressed higher levels of IFN-γ, IL-17, and IL-10, in comparison with Tm-infected TLR2^+/+^ mice (Figure 4B). In addition, we observed that Tm-infected TLR2^−/−^ mice exhibited a higher percentage of splenic CD25^+^Foxp3^+^ Tregs at both 24 h and 7 days p.i. (Figure 5).

### Expression of TLR2 by peripheral blood granulocytes and monocyte subsets is augmented in HH-Tm-infected mice

To evaluate the immune response in a less resistant host, we employed alloxan-induced HH Swiss mice infected with Tm, as previously described (Venturini et al., 2011). We first evaluated the relevance of TLR2 in this context and, subsequently, its impact on the development of infectious processes and induction of the inflammatory response.

**Figure 6:**
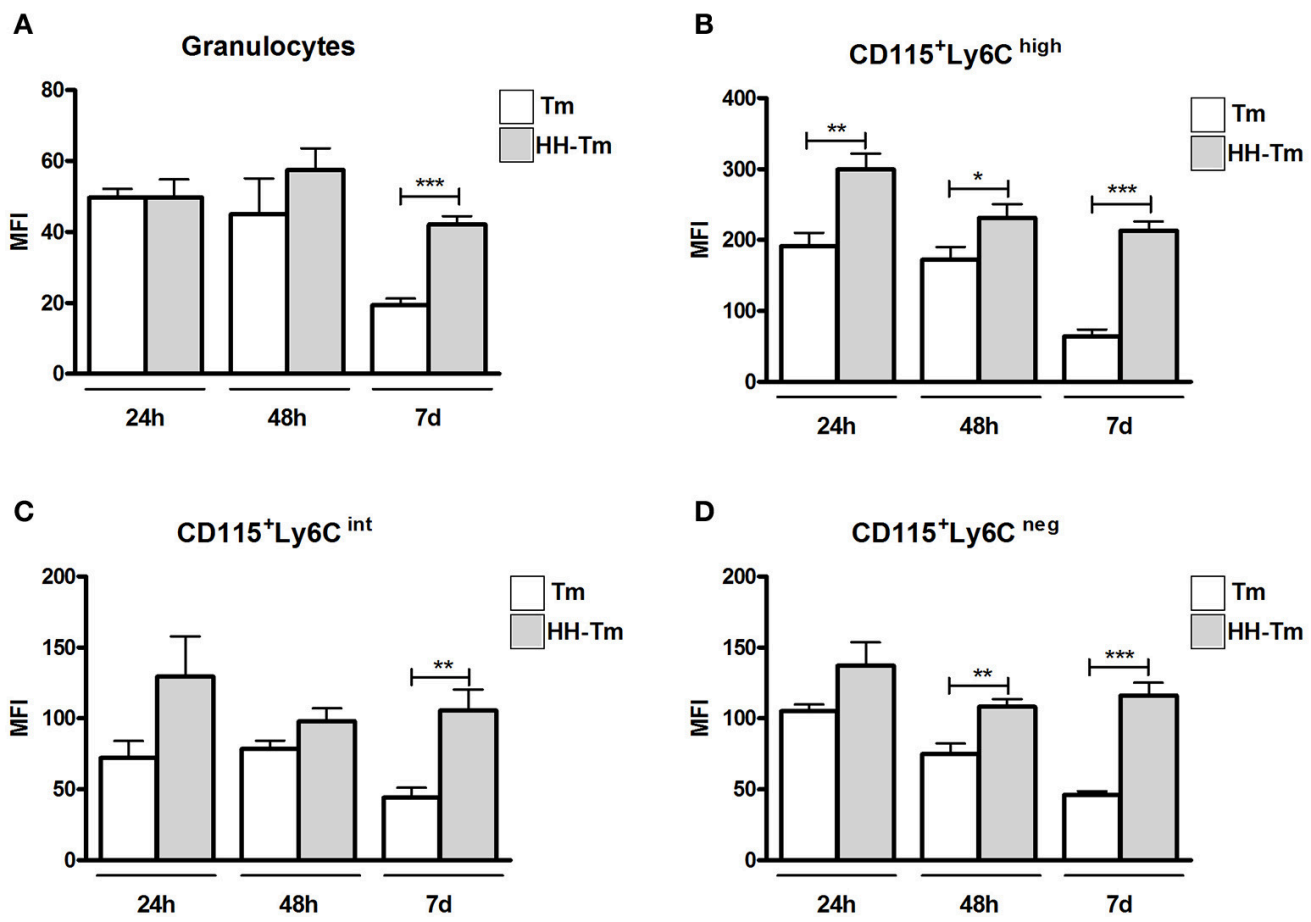
**Expression of TLR2 in peripheral blood granulocytes and monocyte subsets from Swiss mice. (A)** Granulocytes and monocyte subsets **(B–D)** of Tm-infected and HH-Tm mice evaluated at 24 and 48 h, and 7 days, after infection. The results are expressed as means ± SEM (Unpaired *t*-test; ^*^P < 0.05, ^**^P < 0.01, ^***^P < 0.001; *n* = 5–6).

After 24 and 48 h p.i., granulocytes from both Tm and HH-Tm Swiss mice showed similar levels of TLR2 expression (Figure 6A); however, on day 7, granulocytes from HH-Tm mice exhibited higher TLR2 expression than those of Tm mice (Figure 6A). Inflammatory CD115^+^Ly6C^high^ monocytes from HH-Tm mice demonstrated higher TLR2 expression than those from Tm mice at all experimental time-points (Figure 6B). Furthermore, on day 7, HH-Tm mice showed higher expression of TLR2 by intermediate CD115^+^Ly6C^int^ monocytes (Figure 6C) and at 48 h and 7 days, levels of TLR2 were elevated in resident CD115^+^Ly6C^neg^ monocytes (Figure 6D).

During the pilot study, we did not observe alteration in the expression of TLR-2 of non-infected HH mice in the early stage of HH condition (1–2 weeks). High expression of TLR-2 was observed in the late stage of HH (3–4 weeks) (data not shown).

### HH-Tm TLR2^−/−^ mice exhibit improved fungal clearance

**Figure 7:**
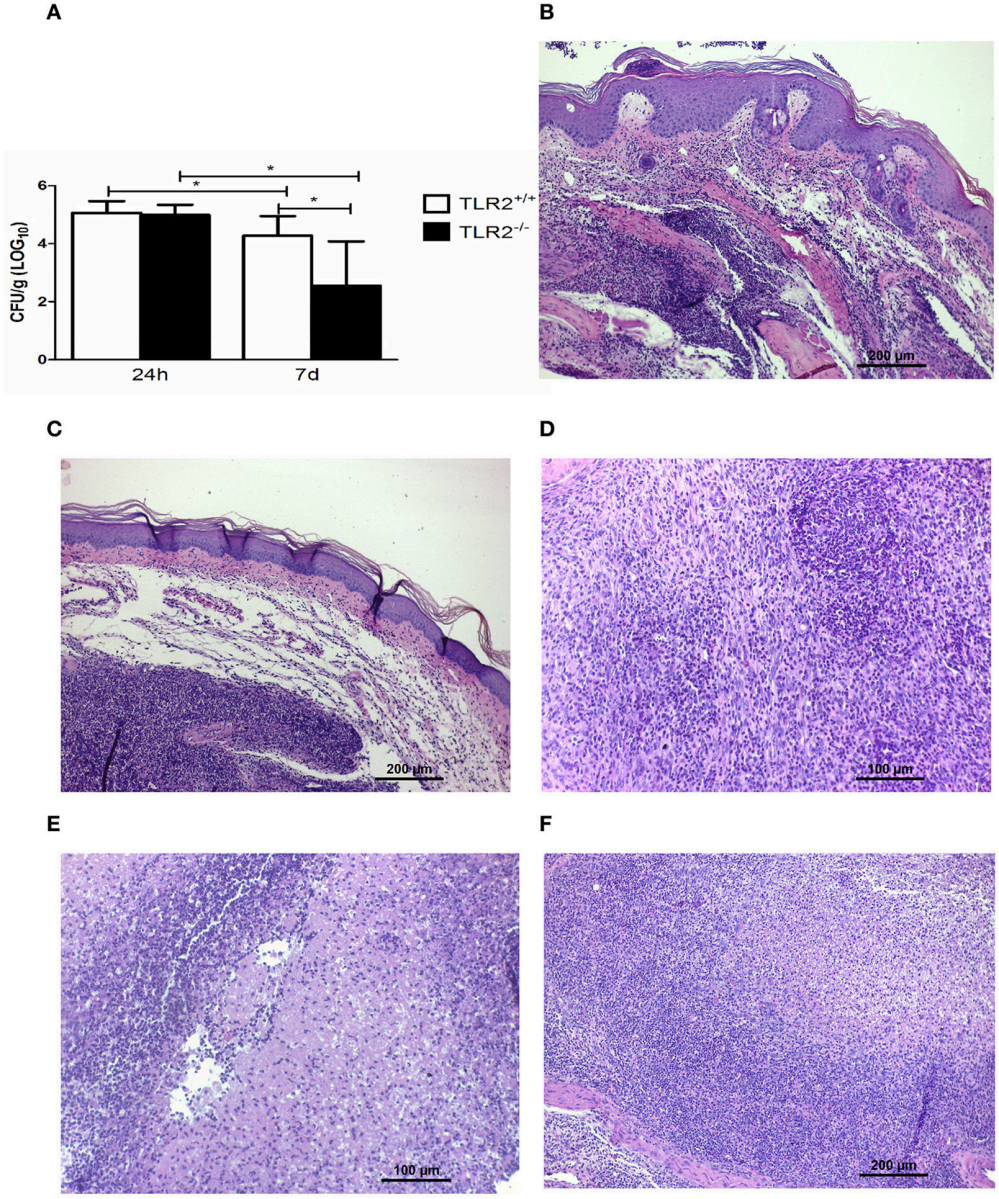
**The dynamics of fungal infection in HH-Tm TLR2^**+/+**^ and TLR2^**−/−**^ mice. (A)** Recovery of viable fungi from footpads of HH-Tm mice 24 h and 7 days p.i. Results are expressed as means ± SD. Unpaired *t*-test: ^*^P < 0.05. **(B)** Footpad of a TLR2^+/+^ HH infected mouse 24 h p.i. exhibiting the initial inflammatory pattern characterized by intense polymorphonuclear infiltrate and edema (HE). **(C)** Footpad of a TLR2^−/−^ HH infected mouse 24 h p.i. exhibiting the initial inflammatory pattern characterized by intense polymorphonuclear infiltrate and edema (HE). **(D)** Footpad of a TLR2^+/+^ HH infected mouse on day 7 p.i. showing a mixed inflammatory infiltrate with a small number of differentiated macrophages and an intense influx of neutrophils (HE). **(E)** Footpad of a TLR2^+/+^ HH infected mouse showing abscess formation and necrosis (HE). **(F)** Footpad of a TLR2^−/−^ HH infected mouse on day 7 p.i., showing more organized granulomas, with a small number of neutrophils, and the presence of modified macrophages (HE).

HH TLR2^+/+^ and HH TLR2^−/−^ mice were infected with Tm and evaluated after 24 h and 7 days. Both groups showed the same level of fungal load in the footpad at 24 h p.i. (Figure 7A); however, on day 7, HH-Tm TLR2^−/−^ mice exhibited a lower fungal load than HH-Tm TLR2^+/+^ mice (Figure 7A).

Histopathological investigation of the inoculation site at 24 h p.i. revealed the same initial inflammatory patterns in both groups, characterized by an intense polymorphonuclear infiltrate and edema (Figures 7B,C). On day 7, a mixed inflammatory infiltrate with a small number of macrophages, an intense influx of neutrophils, abscess formation, and necrosis was observed in HH-Tm TLR2^+/+^ (Figures 7D,E). In contrast, footpads of HH-Tm TLR2^−/−^ mice on day 7 showed more organized granulomas, characterized by a lower influx of neutrophils, and the presence of macrophages and rare epithelioid cells (Figure 7F); in addition, necrotic areas were scarce in these lesions.

### The absence of TLR2 increases the cytotoxicity of macrophages and the regulatory immune response

Next, we investigated the macrophage activity of HH-Tm TLR2^+/+^ and TLR2^−/−^ mice. Both unstimulated and TmExo-stimulated PAC from HH-Tm TLR2^−/−^ mice demonstrated higher production of H_2_O_2_ than those from TLR2^+/+^ HH-Tm mice (Figure 3C). Similarly, TmExo-stimulated PAC from these mice exhibited increased production of TNF-α (Figure 3C), while IL-10 was lower in both unstimulated and TmExo-stimulated PAC from HH-Tm TLR2^−/−^ mice compared with those from TLR2^+/+^ HH-Tm mice (Figure 3C). No differences between the two groups were observed in the production of IL-1β (Figure 3C).

In spleen cell culture, no differences were observed between the HH-Tm TLR2^+/+^ and TLR2^−/−^ groups in the production of IFN-γ or IL-17 (Figure 4C). However, the spontaneous production of IL-10 (Figure 4C) and percentage of CD25^+^Foxp3^+^ Treg cells (Figure 5) were higher in TLR2^−/−^, than in TLR2^+/+^, HH-Tm mice.

## Discussion

Although dermatophytosis is one the most prevalent infections in human and domestic animals (Havlickova et al., 2008), the dermatophyte-host relationship, particularly in relation to disease pathogenesis, is poorly investigated (Almeida, 2008). Besides, there are increased reports of severe cases of deep dermatophytosis, in which the mechanisms involved in the development of this clinical form is unknown. In the present study, we investigated the influence of TLR2 on immune-related events in experimental deep dermatophytosis using diabetic and non-diabetic mouse models, since diabetes mellitus patients are also affected with this fungal infection (Eckhard et al., 2007).

Here, we demonstrated that, in the setting of hyperglycemia, TLR2^+/+^ mice triggered increased pro-inflammatory, but reduced fungicidal activity compared with TLR2^−/−^ mice. We also found that the expression of TLR2 was higher when the infection was associated with the HH condition (HH-Tm group) compared with the group that was infected, but the HH condition not induced. Furthermore, we demonstrate that the presence of TLR2 promotes reduced production of H_2_O_2_ and affects Treg expansion. TLR2 expression was associated with delayed fungal clearance and tissue repair. These mice exhibited severe injuries due to intensive polymorphonuclear inflammatory infiltrate and necrotic areas. In addition, the absence of TLR2 generated a regulatory response, with Treg expansion, increased production of H_2_O_2_ and IL-10 by peritoneal adherent cells, and decreased production of TNF-α. Similar to our results, Devaraj et al. (2011) verified attenuation of pro-inflammatory responses in diabetic TLR2^−/−^ mice. These authors used a model of diabetes induced using the drug streptozotocin and identified decreased NFkB and MyD88 activity, along with IRAK-1 phosphorylation, leading to a decrease in circulating chemokines and cytokines in TLR2^−/−^, compared with wild-type, mice. Our findings, together with those of these authors, suggest that in a diabetes mellitus environment, TLR2 acts to promote a pro-inflammatory response. Thus, studies aiming to block TLR2, as a therapeutic target to prevent complications caused by Diabetes mellitus, will be important.

In healthy mice, Venturini et al. (2012) demonstrated that Tm infection triggers acute inflammation followed by a granulomatous reaction, characterized by a Th1-polarized immune response and IL-10-mediated immune regulation (Venturini et al., 2012). Our findings in the present study demonstrate that TLR2 has a significant role in dermatophytic infection, since its expression is higher in infected, than non-infected, mice. In particular, CD115^+^Ly6C^high^ monocytes exhibited high TLR2 expression, compared to other monocyte subsets. Inflammatory monocytes (CD115^+^Ly6C^high^) are quickly recruited to infected tissues, and their recruitment during dermatophytec infection has been recently reported by our group (Fraga-Silva et al., 2015). Over the course of dermatophytic infection, we identified a decrease in TLR2 expression by the monocytes subsets. Given that mice can eliminate this fungus after 14 days of infection (Venturini et al., 2012), the decreased expression of this receptor is as expected during the resolution of the infection.

Our results indicate that the absence or presence of the TLR2 receptor did not interfere with fungal clearance in healthy mice; however, the absence of TLR2 was favorable for mounting an early granulomatous response, as demonstrated by the increased number of epithelioid macrophages in TLR2^−/−^ mice, which is indicative of a typical Th1 response and efficient immune clearance of dermatophytosis. Indeed, the absence of TLR2 promoted increased production of the Th1 and Th17 cytokines, and an expansion of Tregs, while the presence of TLR2 resulted in insufficient production of IFN-γ, less epithelioid cells at the site of inoculation, and a lower level of H_2_O_2_ production. Considering that on the 14th day p.i. these mice show a typical granulomatous response, with epithelioid macrophages, and are able to eliminate the fungus (Venturini et al., 2012), our results suggest that TLR2 slightly delays the polarization of the Th1 immune response.

The role of the TLR2 receptor in immunity against fungi, and whether it is helpful or not to the host, remains to be elucidated. Netea et al. (2004) reported that the absence of the TLR2 receptor had a beneficial role during *Candida albicans* infection, leading to resistance to disseminated candidiasis, with normal production of pro-inflammatory cytokines, such as TNF-α, IL-1α, and IL-1β, by peripheral adherent cells, and a decrease in the levels of IL-10 and CD4^+^CD25^+^ T lymphocytes. Although disseminated candidiasis promotes an exacerbated inflammatory response, the absence of the TLR2 receptor contributed to a superior, more effectively regulated immune response. In contrast,Loures et al. (2009) demonstrated that the absence of the TLR2 receptor led to an exacerbated inflammatory response, with increased levels of chemokines involved in neutrophil chemotaxis and Th17 type cytokines, and a reduction of Treg expansion during *Paracoccidioides brasiliensis* infection, resulting in more severe lung pathology.

Besides, the important role of TLR2 to interfere in the immune response during dermatophyte infection, especially in the setting of hyperglycemia as demonstrated in the present study, Dectin-1 has emerged as a crucial PRR involved in the immunology of dermatophytosis. It has been described that mutation in CARD9, a key molecule in the C-type lectin receptor (CLR) signaling pathway, was associated to the development of deep dermatophytosis (Lanternier et al., 2013). Yoshikawa et al. (2016) observed that, mice lacking Dectin-1 and/or Dectin-2 showed an inadequate pro-inflammatory cytokine production in response to *T. rubrum* infection, impairing its resolution. Gantner et al. (2003) demonstrated the existence of a cooperative signaling between Dectin-1 and TLR2 for the production of TNF-α by zymosan-stimulated macrophages, but there are no evidences of the involvement with this cooperative during the dermatophytic infection. Therefore, further studies using our model could clarify this cooperative signaling.

In addition, we observed an increased recovery of viable fungi in samples of C57BL/6 mice in comparison to Swiss mice. The evidences of more susceptibility of Swiss mouse strain to Tm are based on old studies. Fischman et al. (1976) observed high prevalence of Tm infection in Swiss mice in an Animal Facility. Schmitt et al. (1963) compared the susceptibility of BALB/c and Swiss mice to dermatophyte and high rate of infection was observed in the Swiss mouse strain. Therefore, our findings include C57BL/6 mouse strain as also more susceptible to Tm.

In summary, our results demonstrate for the first time that: (1) during deep dermatophytic infection, blood monocytes exhibit increased expression of TLR2 and that with HH this expression was exacerbated; (2) TLR2^+/+^ and TLR2^−/−^ mice exhibited similar levels of control of dermatophytic infection; (3) TLR2 modulates the development of a Th1, Th17 adaptive immune response, and Treg numbers in Tm-infected non-HH mice; (4) in an HH environment, TLR2 provokes an intense pro-inflammatory response combined with inefficient fungal clearance. Our study has some limitations; for instance, we did not determine the Tm ligands specific to TLR2 and mechanistic explanations on the role of TLR2 signaling in antigen presentation, phagosome maturation and the development of adaptive immunity; however, our results show, for the first time, the influence of TLR2 in the outcome of experimental dermatophytosis and its impact in the context of a diabetes model during fungal infection. Follow up studies of cell depletion, co-stimulation and TLR2 signaling are needed to test our hypothesis and generate the answers in more detail.

## Author contributions

Conceived and designed the experiments: DA, MA, and JV. Performed the experiments: DA, CM, TF, AS, and AF. Analyzed the data: DA, JV, MG, and VL. Wrote the paper: DA, TF, and JV.

## Funding

This work was supported by grants from the São Paulo Research Foundation (FAPESP) (Grants #2010/16917-2 and #2012/20031-5).

### Conflict of interest statement

The authors declare that the research was conducted in the absence of any commercial or financial relationships that could be construed as a potential conflict of interest.
