# Supplementary material for: TLR2−/− Mice Display Increased Clearance of Dermatophyte Trichophyton mentagrophytes in the Setting of Hyperglycemia
Source: Front Cell Infect Microbiol. 2017 Jan 20;7:8. doi: 10.3389/fcimb.2017.00008 (PMC5248405; doi:10.3389/fcimb.2017.00008)
Supplement: Supplementary file 1 [file Image1.PDF]

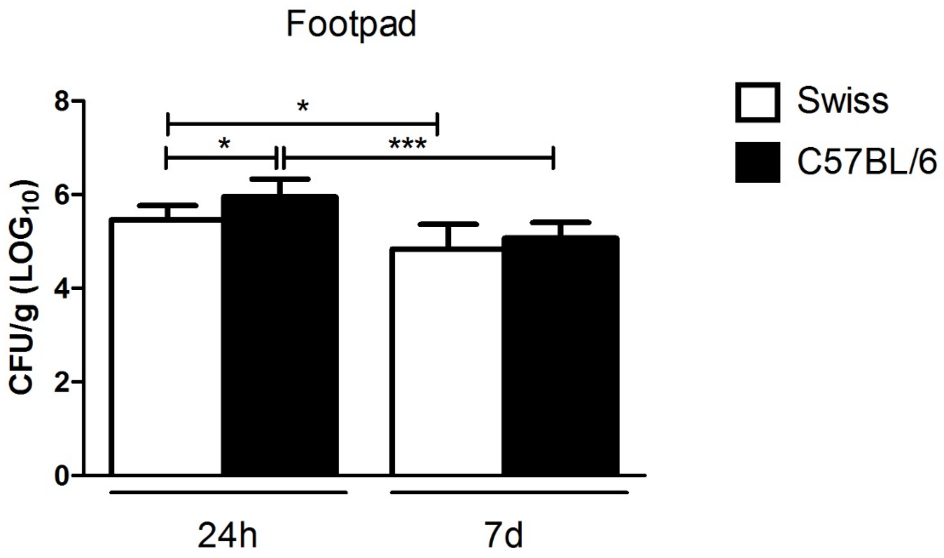

Supplementary Figure 1. Recovery of viable fungi from Swiss and C57BL/6 mice inoculated with *T. mentagrophytes* into the footpad. The mice were evaluated at 24 h and 7 days after inoculation. Results are expressed as means ± SD of CFU/g/log10. (Unpaired t-test: * $P < 0.05$, *** $P < 0.001$).
